# Enhanced Therapeutic Potential of the Secretome Released from Adipose-Derived Stem Cells by PGC-1α-Driven Upregulation of Mitochondrial Proliferation

**DOI:** 10.3390/ijms20225589

**Published:** 2019-11-08

**Authors:** Jaeim Lee, Ok-Hee Kim, Sang Chul Lee, Kee-Hwan Kim, Jin Sun Shin, Ha-Eun Hong, Ho Joong Choi, Say-June Kim

**Affiliations:** 1 Department of Surgery, Uijeongbu St. Mary’s Hospital, College of Medicine, the Catholic University of Korea, Seoul 11765, Korea; lji96@catholic.ac.kr (J.L.); keehwan@catholic.ac.kr (K.-H.K.); 2 Department of Surgery, Seoul St. Mary’s Hospital, College of Medicine, the Catholic University of Korea, Seoul 06591, Korea; ok6201@hanmail.net (O.-H.K.); 0324sjs@naver.com (J.S.S.); hhe49@naver.com (H.-E.H.); hopej0126@gmail.com (H.J.C.); 3 Catholic Central Laboratory of Surgery, College of Medicine, the Catholic University of Korea, Seoul 06591, Korea; 4 Department of Surgery, Daejeon St. Mary’s Hospital, College of Medicine, the Catholic University of Korea, Seoul 34943, Korea; zambo9@catholic.ac.kr

**Keywords:** adipose-derived stem cell, liver regeneration, reactive oxygen species, peroxisome proliferator activated receptor λ coactivator 1α (PGC-1α), secretome

## Abstract

Peroxisome proliferator activated receptor λ coactivator 1α (PGC-1α) is a potent regulator of mitochondrial biogenesis and energy metabolism. In this study, we investigated the therapeutic potential of the secretome released from the adipose-derived stem cells (ASCs) transfected with PGC-1α (PGC-secretome). We first generated PGC-1α-overexpressing ASCs by transfecting ASCs with the plasmids harboring the gene encoding PGC-1α. Secretory materials released from PGC-1α-overexpressing ASCs were collected and their therapeutic potential was determined using in vitro (thioacetamide (TAA)-treated AML12 cells) and in vivo (70% partial hepatectomized mice) models of liver injury. In the TAA-treated AML12 cells, the PGC-secretome significantly increased cell viability, promoted expression of proliferation-related markers, such as PCNA and p-STAT, and significantly reduced the levels of reactive oxygen species (ROS). In the mice, PGC-secretome injections significantly increased liver tissue expression of proliferation-related markers more than normal secretome injections did (*p* < 0.05). We demonstrated that the PGC-secretome does not only have higher antioxidant and anti-inflammatory properties, but also has the potential of significantly enhancing liver regeneration in both in vivo and in vitro models of liver injury. Thus, reinforcing the mitochondrial antioxidant potential by transfecting ASCs with PGC-1α could be one of the effective strategies to enhance the therapeutic potential of ASCs.

## 1. Introduction

Mesenchymal stem cells (MSCs) have shown promising regeneration potential for the liver [1,2,3,4,5,6,7,8]. This is because MSCs are responsible for crucial functions in tissue repair and regeneration in response to damage, reflecting their outstanding characteristics of self-renewal, multilineage differentiation, and appropriate adjustment between quiescence and activation [9]. Of these, self-renewal refers to the ability of MSCs to maintain the stem cell pool for a long time by regenerating cells with the same regenerative potential. Multilineage potential refers to the ability of MSCs to differentiate into specialized cells in each tissue. The adjustment between quiescence and activation means that while MSCs are activated when there is an appropriate stimulus, they return to the quiescent state when the stimulus passes. The combination of these characteristics determines how long MSCs maintain their regenerative potential. Therefore, one should attempt to find a way of balancing as well as optimizing these characteristics of MSCs in the therapeutic application of MSCs into regenerative medicine.

One of the key factors determining these characteristics of MSCs is the appropriate balance between intracellular reactive oxygen species (ROS) production and scavenging them by antioxidant enzymes [9]. In the steady state, ROS levels are maintained at low base levels.

However, when MSCs differentiate into a specific cell type, the content of ROS gradually increases [10]. Thus, maintaining low basal ROS levels in MSCs is expected to be advantageous in letting MSCs persistently have their fundamental characteristics [11,12,13]. Furthermore, excessive increases in ROS levels inevitably lead to the reduction of stem cell function and regenerative potential [13,14,15,16,17,18,19,20,21,22,23]. Therefore, maintaining ROS at low basal levels is a prerequisite for sustaining the optimized potential of MSCs.

Peroxisome proliferator activated receptor λ coactivator 1α (PGC-1α) is a potent regulator of mitochondrial biogenesis and energy metabolism [24,25,26,27]. PGC-1α essentially regulates mitochondrial biogenesis and function by coupling with transcriptional nuclear respiratory factor-1 (NRF-1), mitochondrial DNA transcription factor A (mtTFA), and other metabolic transcriptional nuclear factors [27]. As a result, PGC-1α regulates the expression and activity of mitochondrial antioxidant enzymes [28,29]. Emmeran et al. [29] reported that overexpressing PGC-1α in vascular endothelial cells leads to the increased expression of mitochondrial antioxidant enzymes and thereby the reduction of oxidative stress and cell death. In this study, we first designed adipose-derived stem cells (ASCs) that had been transfected with PGC-1α, and termed them as PGC-ASCs. Subsequently, we intended to determine the therapeutic potential of the secretome released from PGC-ASCs (PGC-secretome) in both in vitro and in vivo models of liver injury.

## 2. Results

### 2.1. In Vitro Validation of PGC-Secretome on Cell Viability and Expression of Various Markers

**Figure 1:**
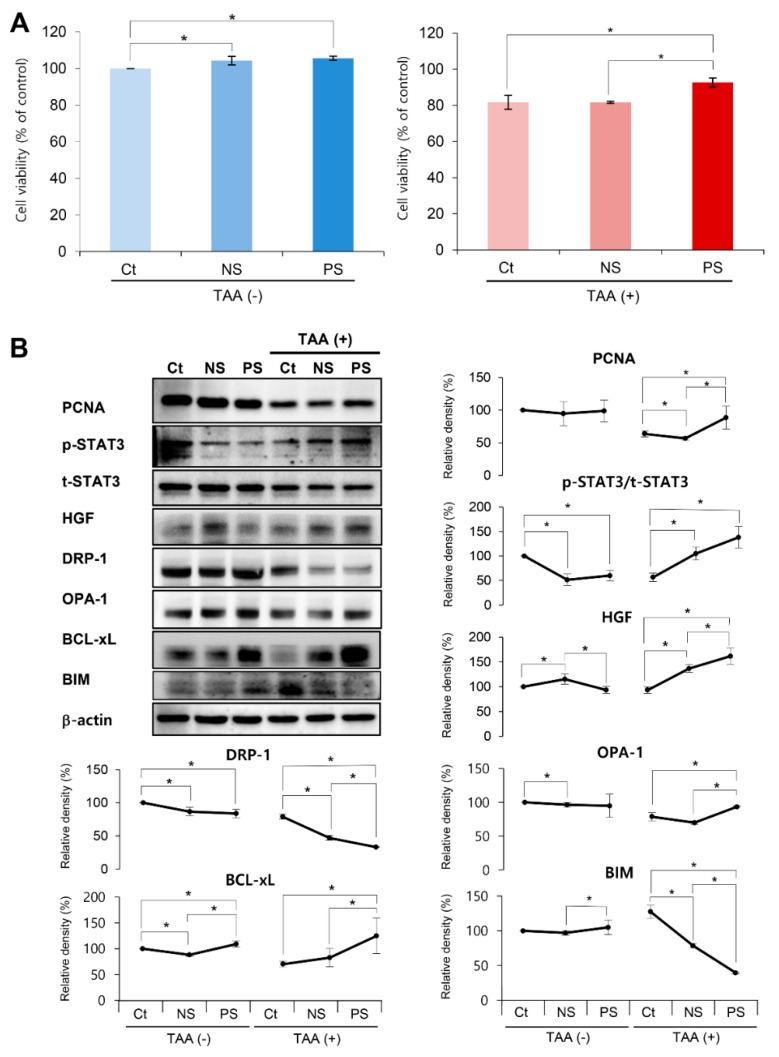
In vitro validation of the effects of the peroxisome proliferator activated receptor λ coactivator 1α (PGC-secretome) on cell viability and expression of various markers. (**A**) Effects of each secretome on the viability of AML12 hepatocytes. Cell viability analysis revealed that the PGC-secretome increased the viability of thioacetamide (TAA)-treated AML12 hepatocytes significantly more than did the control and normal secretome. (**B**) Western blot analysis demonstrating the effects of each secretome on the expression of various markers in AML12 hepatocytes. The markers included those for liver proliferation (p-STAT3, t-STAT3, VEGF, and HGF), mitochondrial fusion (OPA-1), mitochondrial fission (DRP-1), pro-apoptosis (BIM), and anti-apoptosis (Bcl-xL). PGC-secretome significantly increased the expression of markers related to proliferation, mitochondrial fusion, and anti-apoptosis, and significantly decreased the expression of the markers related to mitochondrial fission and pro-apoptosis. Values are presented as mean ± standard deviation of three independent experiments; * *p* < 0.05. Abbreviations: Bcl-xL, B-cell leukemia-extra large; BIM, Bcl-2-like protein 11; Ct, control; DRP-1, dynamin related protein 1; HGF, hepatocyte growth factor; NS, normal secretome; OPA-1, Opa1 mitochondrial dynamin like GTPase; PCNA, proliferating cell nuclear antigen; PS, PGC-secretome; TAA, thioacetamide; VEGF, vascular endothelial growth factor.

A plasmid encoding PGC-1α was transfected into ASCs to produce PGC-1α-overexpressing ASCs. We finally obtained the PGC-secretome from PGC-ASCs after a series of processes, which included centrifugation and filtering, as detailed in the methods section. An in vitro model of liver injury was generated by treating AML12 hepatocytes with the thioacetamide (TAA) hepatotoxin. We first examined the effect of the normal secretome and PGC-secretome on the viability of AML12 hepatocytes. In the control AML12 cells, both secretomes (normal secretome and PGC-secretome) lead to an increased viability in relation to the cells not submitted to secretome. In comparing both secretomes, the effect on the cell viability of PGC-secretome was not significantly different from the normal secretome. However, in the TAA-treated AML12 cells, PGC-secretome increased cells viability not only in comparison with the cells without secretome but also in comparison with the cells treated with normal secretome (*p* < 0.05) (Figure 1A).

We next investigated the effects of each secretome on the expression of various markers in AML12 hepatocytes using western blot analysis. These included markers for liver proliferation (p-STAT3, t-STAT3, vascular endothelial growth factor (VEGF), and hepatocyte growth factor (HGF)), mitochondrial fusion (Opa1 mitochondrial dynamin like GTPase (OPA-1)), mitochondrial fission (dynamin related protein 1(DRP-1)), pro-apoptosis (Bcl-2-like protein 11 (BIM)), and anti-apoptosis (B-cell leukemia-extra large (Bcl-xL)). In TAA-treated AML12 cells, treatment with the PGC-secretome significantly increased the expression of the proliferation-related markers and fusion protein OPA-1, and significantly decreased the expression of fission protein DRP-1 compared with treatment with the normal secretome (*p* < 0.05) (Figure 1B). Treatment with the PGC-secretome also significantly decreased the pro-apoptotic marker BIM and significantly increased an anti-apoptosis markers (Bcl-xL) compared with the treatment with the normal secretome in TAA-treated AML12 cells (*p* < 0.05).

### 2.2. Effects of PGC-Secretome on Mitochondrial ROS Levels

**Figure 2:**
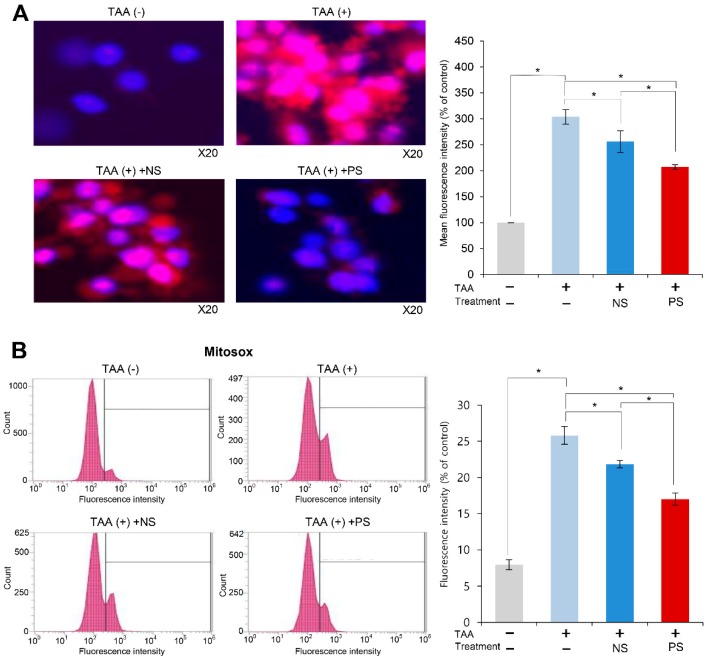
Effects of PGC-secretome on changes of mitochondrial reactive oxygen species (ROS) levels. (**A**) Demonstration of superoxide (ROS) levels by MitoSOX staining (red fluorescence). When MitoSOX Red reagent is oxidized by superoxide (ROS), it produces red fluorescence that accumulates in the mitochondria. Thus, the amount of red fluorescence is proportional to the mitochondrial ROS levels. Whereas TAA-treated AML cells exhibited the highest bright red fluorescence, the secretome treatments significantly decreased it, and of the two secretome groups, PGC-secretome more significantly decreased the fluorescence intensity. (**B**) Quantification of superoxide levels by MitoSOX-based flow cytometry. Whereas TAA-treated AML cells exhibited the highest fluorescence intensity, the secretome treatments significantly decreased it, and of the two secretome groups, PGC-secretome more significantly decreased the fluorescence intensity. Values are presented as mean ± standard deviation of three independent experiments; * *p* < 0.05. Abbreviations: Ct, control; NS, normal secretome; PS, PGC-secretome; TAA, thioacetamide.

We investigated the effect of PGC-secretome on mitochondrial ROS levels using MitoSOX staining. When MitoSOX Red reagent is oxidized by a ROS, such as superoxide, it produces red fluorescence that accumulates in the mitochondria. Therefore, the fluorescence intensity (red fluorescence) is proportional to the mitochondrial ROS levels. Whereas TAA-treated AML cells exhibited the highest bright red fluorescence, the secretome treatments significantly decreased it (*p* < 0.05). Of the two secretome groups, PGC-secretome more significantly decreased the fluorescence intensity (*p* < 0.05) (Figure 2A). Subsequently, we quantified the red fluorescence accumulated in the mitochondria by flow cytometry. Whereas TAA-treated AML cells exhibited the highest fluorescence intensity, the secretome treatments significantly decreased it (*p* < 0.05), and of the two secretome groups, PGC-secretome more significantly decreased the fluorescence intensity (*p* < 0.05) (Figure 2B).

### 2.3. Effects of PGC-Secretome on Liver Regeneration in Partially Hepatectomized Mice

**Figure 3:**
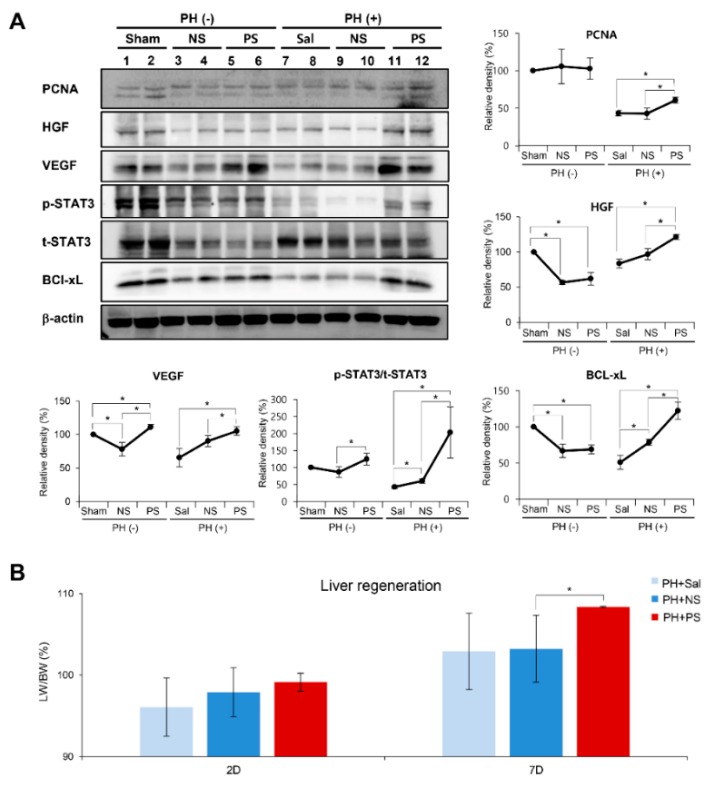
In vivo effects of PGC-secretome on liver regeneration in partially hepatectomized mice. (**A**) (Right) Western blot analysis of the liver at the 7th day following each treatment. The markers studied in the western blot analysis included those for liver regeneration (PCNA, HGF, VEGF, p-STAT3, and t-STAT3) and anti-apoptosis (Bcl-xL). The PGC-secretome group displayed a significant increase in the markers for liver regeneration and anti-apoptosis compared to the levels in the normal secretome group. (Left and Below) Relative densities of the markers in each group. (**B**) Liver regeneration rate (%) based on the ratio of liver weight to body weight (LW/BW) on days 2 and 7 after 70% partial hepatectomy. The PGC-secretome group displayed the highest LW/BW ratios on day 7 after partial hepatectomy. Values are presented as mean ± standard deviation of three independent experiments; * *p* < 0.05. Abbreviations: Bcl-xL, B-cell leukemia xL; Ct, control; HGF, hepatocyte growth factor; LW/BW ratio, ratio (%) of liver weight to body weight; NS, normal secretome; PCNA, proliferating cell nuclear antigen; PH, partial hepatectomy; PS, PCG-secretome; VEGF, vascular endothelial growth factor; Sal, saline.

**Figure 4:**
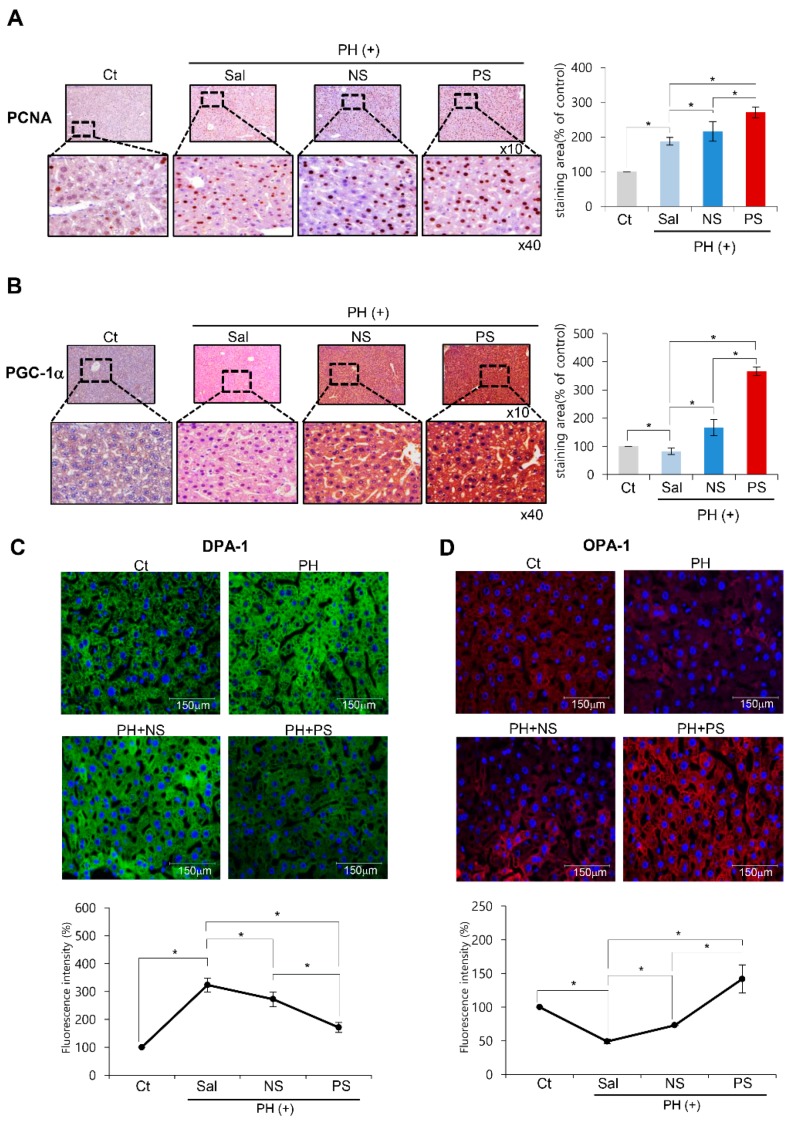
Immunohistochemistry of the liver specimens following each treatment. It shows the immunohistochemistry of PCNA (**A**) and PGC-1α (**B**), and the immunofluorescence of markers related to mitochondrial status (mitochondrial fission marker DPA-1 (**C**) and fusion marker OPA-1(**D**)) using the liver specimens obtained on the 2nd day after each treatment. Percentages of immunoreactive areas were measured using NIH image J and expressed as relative values to those in normal livers. Whereas the PGC-secretome group exhibited the largest immunoreactive area for PGC-1α, OPA-1, and PCNA, it exhibited the smallest immunoreactive area for DRP-1. Values are presented as mean ± standard deviation of three independent experiments; * *p* < 0.05. Abbreviations: Ct, control; DRP-1, dynamin related protein 1; NS, normal secretome; OPA-1, Opa1 mitochondrial dynamin like GTPase; PCNA, proliferating cell nuclear antigen; PGC-1α, peroxisome proliferator activated receptor λ coactivator 1α; PS, PGC-secretome; PCNA, proliferating cell nuclear antigen; PGC-1α, peroxisome proliferator activated receptor λ coactivator 1α; PH, partial hepatectomy; Sal, saline.

To determine the effects of PGC-secretome on liver regeneration, normal secretome or PGC-secretome was injected intravenously into 70% partial hepatectomized mice. On the postoperative 7th day, we attained liver specimens after euthanizing mice, and performed western blot analysis for determining the expression of the markers related to proliferation (PCNA, HGF, VEGF, p-STAT3, and t-STAT3) and anti-apoptosis (Bcl-xL). Western blot analysis revealed a significant increase in the markers for liver regeneration and anti-apoptosis in the PGC-secretome group compared to those in the normal secretome group, as well as the control group (*p* < 0.05) (Figure 3). Liver regeneration was also estimated by the ratio of liver weight to body weight (LW/BW) on day 1, 2, and 7 after 70% partial hepatectomy (Figure 4A) [4,30]. On day 7, the PGC-secretome group showed a significantly higher LW/BW ratio than the other two groups (*p* < 0.05).

### 2.4. Immunohistochemistry and Immunofluorescence of the Liver Specimens 

We attained liver specimens on the postoperative 2nd day, and performed immunohistochemistry of PGC-1α and PCNA, and immunofluorescence of markers related to mitochondrial status (mitochondrial fission marker DPA-1 and fusion markerOPA-1). PGC-1α immunohistochemistry revealed the highest expression of PGC-1α in the PGC-secretome group (*p* < 0.05) (Figure 4A). In the PCNA immunohistochemistry, the PGC-secretome group showed the highest expression of PCNA (*p* < 0.05) (Figure 4B). In DRP-1 and OPA-1 immunofluorescence, the PGC-secretome group showed the lowest expression of fission protein DRP-1 and the highest expression of fusion protein OPA-1 (*p* < 0.05) (Figure 4C–D).

### 2.5. Effects of PGC-Secretome on Systemic Inflammation and Liver Enzymes

**Figure 5:**
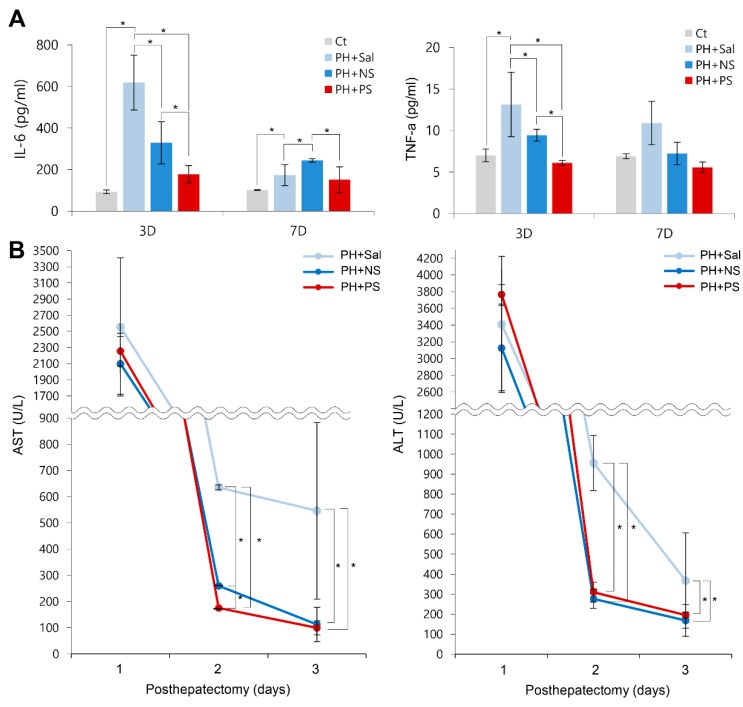
Effects of PGC-secretome on the systemic inflammation and liver enzymes. (**A**) Effects of PGC-secretome on the serum levels of pro-inflammatory cytokines. The PGC-secretome group displayed the most significantly lowered serum levels of interleukin-6 (IL-6, left panel) and tumor necrosis factor-alpha (TNF-α, right panel) compared to the levels in the other groups. (**B**) Effects of PGC-secretome on the serum levels of liver enzymes. Serum levels of aspartate transaminase (AST) and alanine transaminase (ALT) were significantly reduced by the administration of each secretome on days 2 and 3. In comparison of the two secretome groups, the serum levels of AST (left panel) showed a significantly lower values in the PCG-secretome group than in the normal secretome group on day 2. The serum levels of ALT (right panel) were not significantly different between the two groups on days 2 and 3. Values are presented as mean ± standard deviation of three independent experiments; * *p* < 0.05. Abbreviations: ALT, alanine transaminase; AST, aspartate transaminase; TNF-α, tumor necrosis factor-α.

We compared the serum levels of pro-inflammatory markers (interleukin-6 (IL-6) and tumor necrosis factor-alpha (TNF-α)) in each group (Figure 5A). Although 70% partial hepatectomy significantly increased the serum levels of IL-6 and TNF-α, they were significantly reduced by the administration of each secretome (*p* < 0.05). Of the two secretome groups, the PCM-secretome group showed significantly lower serum levels of IL-6 and TNF-α on days 3 and 7 after administration (*p* < 0.05).

**Figure 6:**
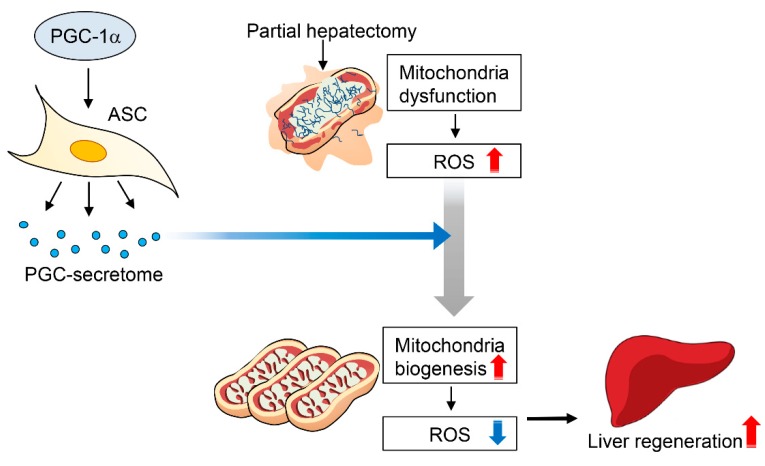
Proposed mechanism of action of PGC-secretome. After partial hepatectomy or liver injury, mitochondrial dysfunction occurs and, subsequently, is followed by rises of intracellular ROS levels. PGC-secretome is expected to promote mitochondrial repair and biogenesis, thereby reducing intracellular ROS levels, resulting in the enhanced liver regeneration.

Finally, we compared the changes in liver enzymes in each secretome group (Figure 5B). On day 1, all the groups exhibited the significantly higher levels of aspartate transaminase (AST) and alanine transaminase (ALT). However, on days 2 and 3, serum levels of AST and ALT were significantly reduced by the administration of each secretome (*p* < 0.05). In comparison of the two secretome groups, the serum levels of AST showeda significantly lower values in the PCG-secretome group than in the normal secretome group on day 2. The serum levels of ALT were not significantly different between the two groups on days 2 and 3. We have provided the possible mechanism of action of PGC-secretome in Figure 6.

## 3. Discussion

PGC-1α is a crucial factor in mitochondrial biogenesis, which is responsible for optimizing stem cell potential by lowering ROS levels through promoting the expression of antioxidant enzymes. This research focused on whether the secretome obtained from PGC-1α-overexpressing ASCs has pronounced regenerative and anti-inflammatory potential compared to the naïve secretome. In the in vitro experiments using TAA-treated AML12 hepatocytes, the PGC-secretome significantly increased cell viability and the expression of the proteins related with proliferation and anti-apoptosis. MitoSOX-based flow cytometry demonstrated that the PGC-secretome also significantly decreased the mitochondrial ROS levels compared to the normal secretome. In the in vivo experiments using 70% partially hepatectomized mice, PGC-secretome injections significantly increased the expression of the markers related to proliferation and anti-apoptosis in the livers compared to the effects of the normal secretome. In addition, PCNA immunohistochemistry and LW/BW ratio analyses confirmed that PGC-secretome injections led to significantly higher liver regeneration than did normal secretome injections. Moreover, the PGC-secretome significantly reduced serum levels of pro-inflammatory cytokines (IL-6 and TNF-α) and liver enzymes (AST and ALT) compared to the normal secretome. Taken altogether, the results herein demonstrate that the PGC-secretome has higher antioxidant, anti-inflammatory, and higher regenerative properties than the naïve secretome in both in vitro and in vivo models of liver injury.

MSCs exhibit anti-inflammatory, reparative, and immunomodulatory properties that are mediated by the secretome [31,32,33]. However, cell therapy using naïve MSCs has various limitations, including low proliferation rate, relatively lower potency, and gradual loss of stemness during ex vivo expansion [34,35,36,37]. MSCs have a relatively high plasticity, and thus can be appreciably changed in terms of their characteristics according to the culturing conditions. Therefore, optimizing the culture conditions is desirable to attain a sufficient number of efficient MSCs. Culture conditioning can be largely divided into physicochemical conditioning and genetic manipulation. In this study, PGC-1α-overexpressing ASCs were utilized as a way of genetically manipulating MSCs. In addition, the PGC-secretome was utilized as the therapeutic material instead of PGC-ASCs to reduce the burden on future clinical applications.

Physicochemical conditioning is a way of achieving large amounts of effective MSCs by adjusting the physicochemical environment that MSCs are cultured in. Physicochemical conditioning includes a number of preconditioning techniques using hypoxia [5,6], hydrogen peroxide [38], lipopolysaccharide [4], serum deprivation [39], SDF-1 [40], and transforming growth factor-β [41]. By contrast, genetic manipulation is a way of potentiating MSCs mostly by overexpressing cytoprotective genes. In one study, cardiac function was reported to be improved by transplantation of Akt-overexpressing MSCs [42]. In addition, MSCs overexpressing heat shock protein 20 improved the engulfment of MSCs in ischemic conditions [43]. Likewise, generation of antioxidant enzymes was enhanced by transfecting MSCs with nuclear factor erythroid 2-related factor 2 (Nrf2) [44]. The present study provides further evidence of the strengthened functions of MSCs through genetic manipulation. The present study is in line with such achievements of enhancing the potency of MSCs using genetic manipulation. Moreover, the present study is the first report, as far as we know, to attain the higher regenerative and anti-inflammatory characteristics as well as antioxidant activities by transfection of PGC-1α into ASCs

PGC-1α is essential for the activation of the complete program of mitochondriogenesis as well as cellular respiration. In general, mitochondrial activity in MSCs is regulated by mitochondrial biogenesis mediated by mTOR complex 1 (mTORC1) [9]. mTORC1 enhances mitochondrial biogenesis by inhibiting eIF4E-binding proteins and by increasing the expression of nucleus-encoded mitochondrial proteins and PGC-1α, the key coordinator of mitochondrial biogenesis [45]. A recent study described that mitochondrial biogenesis in stem cells was also promoted by the upregulation of PGC-1α [46]. In a model of Huntington’s disease, mitochondrial dysfunction was caused by the inhibition of PGC-1α by a mutant form of the huntingtin protein; overexpression of PGC-1α rescued cells from the deleterious effect of huntingtin, demonstrating the significance of PGC-1α [47]. PGC-1α contributes to the mitochondrial biogenesis by integrating and regulating the activity of multiple transcription factors, such as NRF-1, peroxisome proliferator activated receptor alpha (PPARα), and mitochondrial transcription factor A. Overexpressing PGC-1α leads to the increased expression of mitochondrial antioxidant enzymes and thereby the reduction of oxidative stress and cell death. [29] By contrast, dysregulation of mitochondrial biogenesis leads to increased oxidative phosphorylation and increased ROS levels, eventually leading to stem cell exhaustion [46,48]. In this study, it can be postulated that PGC-1α transfection into ASCs leads to the optimization of the essential components of stem cell functions, such as self-renewal and differentiation, by lowering ROS levels.

Within this study, the PGC-secretome was utilized instead of PGC-ASCs due to limitations in cell-based therapy. Cell-based therapy has the risks of senescence-related genetic instability, limited cell survival in the in vivo environment, and immunological rejection. Particularly, the major obstacle hindering the widespread adaptation of stem cells is that one cannot completely exclude the possibility of malignant transformation [49,50]. In this study, the secretome released from ASCs was highlighted, since it is responsible for a substantial proportion of therapeutic potentials of MSCs. The secretome is the sum of all products secreted by stem cells and includes chemokines, cytokines, and growth factors. As secretome research has progressed, it has been demonstrated that secretome contains not only protein components but also vesicles that contain non-protein components, such as DNAs, lipids, messenger RNA, and microRNAs [49]. The vesicles are termed extracellular vesicles, and comprise exosomes (30–100 nm), microvesicles (100 nm–1 μm), and apoptotic bodies (1–5 μm) depending on their size. The secretome of MSCs basically have anti-inflammatory and immunomodulatory properties [51,52,53,54,55,56]. The present data indicate that the PGC-1α transfection reinforces ASCs to release the secretome with higher anti-inflammatory, reparative, and antioxidative properties than those of naïve secretome.

In conclusion, the superior therapeutic potential of the PGC-secretome over the naïve secretome was demonstrated in terms of higher antioxidant, anti-inflammatory, and regenerative potentials. PGC-secretome increased cell viability, promoted expression of the proliferation-related markers, and significantly reduced mitochondrial ROS levels in TAA-treated AML12 cells. In the in vivo experiments using 70% partially hepatectomized mice, PGC-secretome injections significantly enhanced liver regeneration and increased the expression of the proteins related to proliferation and anti-apoptosis in the liver compared to normal secretome injections. Moreover, the PGC-secretome significantly reduced serum levels of pro-inflammatory cytokines (IL-6 and TNF-α) and liver enzymes (AST and ALT) compared to the normal secretome. Therefore, the use of secretome released from PGC-1α-overexpressing ASCs could be an effective strategy to enhance the therapeutic potentials of ASCs.

## 4. Materials and Methods

### 4.1. Cell Culture

Human ASCs were kindly donated by Hurim BioCell Co. Ltd. (Seoul, Korea) (IRB number 700069-201407-BR-002-01). ASCs were plated into a culture flask in low-glucose Dulbecco’s Modified Eagle’s Medium (DMEM) (Thermo Fisher Scientific, Carlsbad, CA, USA) supplemented with 10% fetal bovine serum (FBS) (Thermo), 100 U/mL of penicillin (Thermo), and 0.1 mg/mL of streptomycin (Thermo). A non-tumorigenic AML12 mouse hepatocyte cell line (CRL-2254), was purchased from American Type Culture Collection (ATCC; Manassas, VA, USA). AML12 cells were maintained in DMEM/F12 (DMEM/Ham’s F-12; Thermo). The medium was supplemented with 10% fetal bovine serum (FBS) (GibcoBRL, Calsbad, CA, USA), 1% antibiotics (Thermo), 1 × insulin-transferrin-selenium-G (ITS) supplement (Invitrogen, Calsbad, CA, USA), and 40 ng/mL dexamethasone (Sigma–Aldrich, St. Louis, MO, USA). Cells were incubated at 37 °C in a humidified chamber containing 5% carbon dioxide.

### 4.2. Attainment of PGC-Secretome

ASCs were grown in 100 mm cell dishes (Corning Glass Works, Corning, NY, USA). After reaching 70–80% confluence, the ASCs were transiently transfected with 1 μg pcDNA-PGC-1α. The PGC-1α plasmid was purchased from OriGene Technologies (Rockville, MD, USA). After 24 h, 1.0 × 10^6^ ASCs were cultured in 5 mL serum-free low-glucose DMEM for 24 h. Therefore, to obtain 0.2 mL of secretome from 1.0 × 10^6^ ASCs, the conditioned media were concentrated 25-fold using ultra filtration units with a 3-kDa molecular weight cutoff (Amicon Ultra-PL 3; Millipore, Bedford, MA, USA). We then injected 0.1 mL of secretome per mouse. This means that one mouse was injected with the secretome obtained from 5 × 10^5^ ASCs. In this study, normal secretome refers to the secretome obtained from empty vector-transfected ASCs, and PGC-secretome refers to the secretome obtained from pcDNA-PGC-1 α transfected ASCs.

### 4.3. Cell proliferation Assay

Cell proliferation of AML12 mouse hepatocyte cell line were evaluated using EZ-Cytox Cell Proliferation Assay kit (Itsbio, Seoul, Korea) according to the manufacturer’s instructions.

### 4.4. Design of Animal Study

Animal studies were carried out in compliance with the guidelines of the Institute for Laboratory Animal Research, Korea. We used five-week male BALB/c mice (Orient Bio, Seongnam, Korea) in this study. We compared the effects of normal secretome and PGC-secretome in an in vivo model of 70% partial hepatectomy (PH) [57]. Mice were largely divided into two groups: control mice (*n* = 42) that underwent a sham operation and PH mice that underwent 70% PH (*n* = 42). Subsequently, the control mice and the PH mice were intravenously (using tail vein within 1 h after PH) injected with normal saline (*n* = 14), normal secretome (*n* = 14), and PGC-secretome (*n* = 14), respectively. The mice were euthanized to obtain blood and liver samples at specific time points.

### 4.5. Western Blot Analysis

The AML12 cells and liver specimens obtained from the mice were lysed using the EzRIPA Lysis kit (ATTO Corporation; Tokyo, Japan), and quantified by Bradford reagent (Bio-Rad, Hercules, CA, USA). Proteins were visualized by western analysis using the following primary antibodies (1:1000 dilution) from Cell Signaling Technology (Beverly, MA, USA) and then with HRP-conjugated secondary antibodies (1:2000 dilution) from Vector laboratories (Burlingame, CA, USA). Specific immune complexes were detected using the Western Blotting Plus Chemiluminescence Reagent (Millipore, Bedford, MA, USA).

Primary antibodies against proliferating cell nuclear antigen (PCNA), phosphorylated-signal transducer and activator of transcription 3 (p-STAT3), STAT3, hepatocyte growth factor (HGF), vascular endothelial growth factor (VEGF), B-cell leukemia-extra large (Bcl-xL), dynamin related protein 1 (DRP-1), and β-actin were all obtained from Cell Signaling Technology (Beverly, MA, USA), and Opa1 mitochondrial dynamin like GTPase (OPA-1) was obtained from Santa Cruz biotechnology (Santa Cruz, CA, USA). Horseradish peroxidase (HRP)-conjugated secondary antibody were obtained from Cell Signaling Technology (Beverly, MA, USA).

### 4.6. Serology Test and ELISA

Blood samples were collected from each mouse. We measured the concentrations of markers for liver injury, such as aspartate transaminase (AST) and alanine transaminase (ALT) using an IDEXX VetTest Chemistry Analyzer (IDEXX Laboratories, Inc., Westbrook, ME, USA). The concentrations of mouse interleukin (IL)-6 and tumor necrosis factor (TNF)-α were measured by sandwich enzyme-linked immunosorbent (ELISA) assay (Biolegend, San Diego, CA, USA) according to the manufacturer’s instructions.

### 4.7. Immunohistochemistry

For immunohistochemical analysis, formalin-fixed, paraffin-embedded tissue sections were deparaffinized, rehydrated in an ethanol series, and subjected to epitope retrieval using standard procedures. Antibodies against PCNA (Cell Signaling Technology), PGC-1α (Novus Biologicals, Centennial, CO), DRP-1 (Cell Signaling Technology), and OPA-1 (Santa Cruz biotechnology) were used for immunochemical staining. The samples were then examined under a laser-scanning microscope (Eclipse TE300; Nikon, Tokyo, Japan).

### 4.8. MitoSOX Staining and Flow Cytometry

The AML12 cells were cultured on Lab-Tek chamber slides (Thermo Fisher Scientific, Waltharm, MA). The cells were treated with control secretome and PGC-secretome for 24 h, respectively. Subsequently, The AML12 cells were stained with 10 μM MitoSOX reagent at 37 °C for 10 min. The mitochondrial ROS levels were determined using a fluorescence imaging system (EVOS U5000; Invitrogen, CA, USA). For the quantification of mitochondrial ROS levels in the cells, we performed MitoSOX-based flow cytometry. After incubating cells with MitoSOX reagent for 10 min in the dark at 25 °C, fluorescence intensity of the cells was analyzed using an Attune NxT Acoustic focusing cytometer (Thermo fisher scientific, MA, USA).

### 4.9. Statistical Analysis

All data were analyzed with SPSS 11.0 software (SPSS Inc., Chicago, IL, USA), and are presented as mean ± standard deviation (SD). Statistical comparison among groups was determined using the Kruskal–Wallis test followed by the Dunnett’s test as the post hoc analysis. Probability values of *p* < 0.05 were regarded as statistically significant.

## Abbreviations

ALT	alanine transaminase
ASC	adipose-derived stem cell
AST	aspartate transaminase
Bcl-xL	B-cell leukemia-extra large
BIM	Bcl-2-like protein 11
DRP-1	dynamin related protein 1
HGF	hepatocyte growth factor
OPA-1	Opa1 mitochondrial dynamin like GTPase
PCNA	proliferating cell nuclear antigen
PGC-1α	peroxisome proliferator activated receptor λ coactivator 1α
PH	partial hepatectomy
ROS	reactive oxygen species.
TAA	thioacetamide
TNF-α	tumor necrosis factor- α
VEGF	vascular endothelial growth factor
